# Dental Hints Department

**Published:** 1907-05

**Authors:** B. H. Teague

**Affiliations:** Aiken, S. C.


### ...DENTAL HINTS DEPARTMENT...

CONDUCTED BY B. H. TEAGUE, D. D. S., AIKEN, S. C., TO WHOM ALL CONTRIBUTIONS TO THIS DEPARTMENT SHOULD BE SENT

Porcelain should be the only proper inlay material used. A good gold or amalgam filling is superior to a gold inlay.

For facial neuralgia, a small quantity of oil of sweet birch rubbed over the nerve gives great relief.—Era.

Aluminum Washers.—Aluminum plate makes washers where you want a close joint. It is better than lead on a vice where you wish to protect what you are gripping.—A. G. Knight, Commonwealth Dental Review.

Cleaning Files.—When a vulcanite file becomes clogged with rubber and plaster, wrap absorbent cotton around it and saturate the cotton with chloroform. In about ten minutes it can be cleaned perfectly, using a stiff brush wheel on the lathe.—V. P. Perisho, Dental Review,

Gold Inlay.—After placing matrix of gold roughly, it was packed full of moss-fibre gold, removed and solder placed over the gold; then replaced, re-adapted to the margins with burnishers and solder placed to proper contour. Investment unnecessary.—Clinic by W. O. Fehman, Northwestern Dental Journal.

A Haemostatic.—Potassium permanganate is a valuable haemostatic used in the form of a paste made by mixing it with 4 per cent. of vaselin. Dry thoroughly the part to which it is to be applied. Keep in an air-tight receptacle when not in use.—Brief.

Science to Make Dentures.—I claim that it takes more science to make a set of teeth than any other work, and a good dentist can always be recognized by his ability to do that class of work.—C. V. Vignes, Dental Headlight.

Remedy for a Warped Plate.—A rubber plate which has been bent out of shape will return to its original shape if dropped in hot water and kept there a few moments.—Pacafic Dental Gazette.

Fatal Hemorrhage Following Tooth Extraction.—Death was the result of having eight teeth extracted, in the case of George E. Stevens, a tobacco grower of East Hartford, Conn. Stevens' teeth ached so severely that he had eight of them taken out. He lost so much blood that he became very weak and finally died of exhaustion. He was 28 years old.—Hartford Telegram.

To prevent "etching" of facings when using twenty per cent. platinum solder, paint the surface with a creamy solution of magnesium carbonate before investing—Dr. A. E. Maneson, Review.

A new alloy, which serves electricians as a substitute for platinum, consists of 16½ ounces of silver, 4½ pounds of nickel, ½ ounce of bismuth and 53 pennyweights of gold. The cost is about one-thirtieth of that of platinum.—Era.

There should be a dentist on every hospital staff, on the medical staff of every home for the orphan or the aged and of every other public or eleemosynary institution. I will go further and state that every institution that requires a physician should also have a dentist.—Dr. F. W. Wiseman, Era.

To Etch the Surface of a Gold Inlay.—When the inlay is ready to set, dip the part to which the cement is to adhere in mercury, coating it evenly, spreading it around with the aid of a pellet of moist cotton held in the fluid. Then invert over an alcohol flame and drain off the mercury, which will leave a rough, crystalline surface.—C. I. Hadley, Dental Review.

Sensitive Cavities.—Zinc iodine crystals 1½ gr., iodine crystals 2 grs., make a solution of the above in glycerin. Wind a small pellet of cotton on a broach, dip in the solution and apply to the decay. To remove the stain use hydrogen dioxide. I have found this very effective.—E. M. S. Fernandez, Dental Review.

Sterilization of Dentures.—Sulfurous acid will absolutely deodorize and disinfect a denture and not merely cover the odor of the plate that has been worn in the mouth. Place a few drops in a little water and immerse the case in the solution at night and cleanse with soap and brush in the morning.—J. Kennerly, British Dental Journal.

Sensitive Cervical Margins.—If bicarbonate of soda is incorporated in the tooth powder used by the patient, sensitiveness will be relieved and he will be enabled to thoroughly masticate, bringing about a normal condition of the salvia and the alkaline powder will not be long required.—D. Spalding, Dental Register.

The Age for Regulating.—I have continually advocated the early regulation of the teeth—that is while they are erupting and the bone and jaws are in the formative stage. If the arch needs expansion it should be done while nature is at the height of activity in the deposit of bone in these parts.—V. H. Jackson, Cosmos.

Hydronaphthol as a Pulp-capping.—To avoid the removal of the layer of softened dentin, which if removed, would propably necessitate the removal of the pulp, mix equal quantitie of hydronaphthol and cement, and place as a capping on the layer of decalcified dentin, allowing it to set. Then proceed with the filling. The hydronaphthol arrests bacterial action.—A. W. McCall, Federal Dental Journal.

Chloroform Water as a Hemostatic.—This is used by Spaak (Journal de Med. September 16, 1906), who finds it superior to all other styptics. It acts with marvelons rapidity, has not the slightest disagreeable taste or odor, is not escharotic, is easily obtainable, and can be made as required. It is not unpleasant when applied, and does not interfere with the surgeon in his operations. Spaak recommends a two per cent. simple solution in water.—Medical Times.

Removal of Warts.—For the removal of warts as well as corns salicylic acid is extremely efficacious. It is best used in the following form:

| | |
|---|---|
| Salicylic acid | 5 |
| Extract of cannabis indica | 1 |
| Collodion | 60 |

This is to be painted on the wart or corn at bedtime, with a camelshair brush. In four or five days the growth may be readily peeled off with a knife, leaving a tender but entirely healthy skin at its site.—Clin. Med.

A Glass Solder.—M. Margot states that an alloy composed of tin 95 parts and zinc 5 parts, which melts at 360 degrees F., adheres to glass tenaciously, and possesses an unalterable metallic luster. An alloy of tin 90 parts and aluminum 10 parts, melting at 572 degrees F., also adheres strongly to glass surfaces. By means of either of these formulæ two glass surfaces can be united as if they were of metallic composition.—Ch. Margot, Le Laboratoire.

If it is found difficult to remove an impression for full superior denture, have the patient close the lips and blow with sufficient force to distend the cheeks, and the impression will drop down, no matter how tight it may have been.—R. C. Traynham, Practical Dental Journal.

Etching Porcelain Inlays.—Hydrofluoric acid makes a smooth etch; white acid makes a frosted etch, to which the cement will tightly adhere. It is prepared by making a saturated solution of ammonium carbonate in hydrofluoric acid, using a lead dish; evaporate to one-half its bulk; add hydrofluoric acid up to its original bulk, and evaporate again to one-half. Keep it in a guttapercha bottle.—Joseph Head, Dental Cosmos.

Cement Fillings.—When conditions indicate the advisability of having a small portion of infected (though not softened dentin) the cavity must be sterilized with a cauterizing, non-staining disinfectant, such as colorless tricresol and formalin, equal parts; all traces of this solution to be removed from the cavity by applications of hot air, or repeated washings with distilled water, before the cement is inserted.—R. H. Welsh, Items of Interest.

Gas and Time Regulator.—Every vulcanizer ought to be equipped with a regulator; not only because this prevents too rapid heating, too high heating, and too long heating, but rather because it removes the necessity for watching the vulcanizer, and—most important—the danger of explosion from neglect in watching.—Stewart J. Spense.

Amalgam Fillings.—For amalgam fillings in first and second molars, gold is indicated to bring the alloy to the required hardness and edge strength. If copper is substituted instead of gold the result is rapid discoloration, whereas gold whitens the alloy and hardens it to any desired extent, and most important of all, gives such a knife-edge water-tightness as seems to meet with no equal.—W. Charles Davis, The Dental Record.

Do Not Varnish Inlays After Setting.—The varnishing of an inlay after setting for the purpose of keeping the moisture from the cement has proved incorrect. The cements that are used to-day in setting inlays are what are called hydraulic cements. We have better success with such cements, for after a proper crystallization has taken place the moisture is immediately allowed to flow over.—W. H. Cudworth, Dental Brief.

"The irritation produced by ulceration at the root of a tooth is usually more liable to interrupt pregnancy than the administration of nitrous oxide and the removal of the tooth or establishing free drainage. The author has had gas given for this purpose and has never seen any bad results."—Dr. M. E. Jordon, Era.

NOVOCAIN.—The especial advantages of novocain are found in the extremely slight toxicity, combined with a great anesthetic power, and the absence of all irritating or pain-producing side action. In 10 per cent. solution it may be used as an application to mucous membrane and is especially recommended for use in dental work. It is frequently combined witn suprarenal preparations.—New Orleans Medical and Surgical Journal.

Where children come with large cavities in molars, but with no pulp exposure as yet, the thorough and often painful (painful if thorough) removal of decay may be avoided if the nitrate of silver treatment is used prior to inserting a filling; and thus our work is durable, while tediousness and pain are largely eliminated, and the child is our friend.—DR. R. B. TULLER, Amer. D. Jour.

PERICEMENTITIS.—If, instead of using equal parts of aconite, iodine, and chloroform, you use this prescription:

Rx. Tinc. Aconiti (rad.)...... ........fluid ounces i

Chloroformi..........................fluid ounces iv

Menthol....................................gr. xx you will get excellent results.—J. P.BUCKLEY, Dental Digest.

WHEN AND WHEN NOT TO EXTRACT.—(a) If not more than three scattered teeth remain, extract them. (b) If two molars or bicuspids remain on each side, do not extract. (c) If only the incisors remain, extract. (d) If four or five teeth remain on one side of the jaw and none on the opposite side extract.—Wes. Dental Journal.

NOVOCAIN.—Novocain is from five to six times less toxic than cocain; it does not irritate in the slightest degree when injected, it is soluble in its own weight of water; it will combine with adrenalin in any proportion; it is readily absorbed by the mucous membranes. It seems very probable that novocain has been destined to rob local anæsthesia of its dangers and to be the ideal substitute of cocain.—HERMAN PRINZ, Dental Era.

Legacy to Hartford Dental Society.—By the will of Mrs, Ella Burr McManus, daughter of the late A. E. Burr, of the Hartford Times, $101,000 is given for benevolent purposes, mostly in Hartford. Provision is made for a memorial to her father. The husband of the testatrix, Dr. James McManus. is to have life use of the estate. Among the bequests are $55,000 to the Humane Society, $20,000 to the American Anti-Vivisection Society and $10,000 to the Dental Society. —New York Herald.

I am not prepared to agree with the practitioners in both the dental and the medical worlds who believe, first, that the mother's dental organs are broken down to supply any fetal demands; second, that the mother suffers from the lack of lime salts after the fetus is supplied; and third, that an unusual susceptibility to caries is necessarily an accompaniment to pregnancy.—Dr. M. E. Jordon, Era.

Formaldehyde.—The great value of formic aldehyde lies in its great power of penetrating tissues, sterilizing as it goes. In teeth which have been treated, and in which every attempt to seal them up is followed by pain and tenderness, a 5 per cent. solution used as a dressing on a wisp of cotton and left in for a week will remedy all that, and the tooth may be filled wiih the assurance that all will be well.—B. J. T. Bennette, Dental Record.

Chloroform Water as Hemostatic.—It acts with marvelous rapidity; it has not the slightest disagreeable taste or odor; it is not escharotic; it is cheap, easily obtainable, and can be made as required; it is not unpleasant to apply and does not interfere with the surgeon in his operations. Spaak recommends a two per-cent. simple solution in water.—Medical Times.

Veneering Vulcanite Plates.—Pack in the usual manner, open flask, and, if there should be all the rubber needed, take a sheet of the vulcanite used for veneer and stretch it until quite thin, and place it over the vulcanite in the flask, close flask, and vulcanize. This can be used to produce beneficial and beautiful vulcanite plates. To line a plate for instance with black rubber, it can be done by stretching a thin sheet of black rubber over the surface of the case, or by painting the plaster model with a solution of black rubber, then pack the flask and vulcanize.—W. J. Robinson, Stomatologist.

Removing Plaster From the Hands.—In removing plaster from the hands after the application of plaster casts, it would be well to remember the fact that syrup of lime is the strongest solution, and that the application of a little sugar to the hands will greatly assist you. The same rule applies to the removal of casts.—Ga. Jour. of Med. and Sur.

Removing Glaze from Carborundum Stones.—To renew carborundum stones that have become glazed from grinding down teeth containing amalgam fillings, place them in a beaker and cover with a fifty per cent. solution of nitric acid, allowing them to remain for two or three hours. Remove and place in a strong solution of sodium bicarbonate for several hours that the acid which has been absorbed by the stones may be neutralized.—W. H. Tweedle, Dental Review.

Sensitive Dentin.—Some time ago Dr. Fossume spoke of the use of "Formalin" for controlling the sensitive spots at the necks of the teeth that are sometimes so troublesome. At that time I questioned its safety, but since trying the method I have found it so satisfactory that I wish to speak of it at this time. The "Formalin" should be used full strength and it must be kept away from the gum. A very short application is sufficient to control entirely a very large proportion of these cases.—H. W. Gillett, October 1906 Journal.

Treatment of Sensitive Palates Prior to the Taking of Plaster Impressions.—In prosthetic work, when your patient has a tender palate and is bothered with retching, to stay such trouble while taking an impression, paint on palate a twenty per cent. solution of potassium bromid with a camels-hair brush, or gargle 5 to 15 gr., or both. In extreme cases give 10 gr. at night, repeat after breakfast, and again, half an hour before impression is taken, give 10 to 15 gr. It greatly diminishes the sensitivity of a tender palate.—H. E. Davis, Dental Era.

Irregularities in the Deciduous Teeth.—During four or five years I have advanced the opinion that the position of deciduous teeth affect the permanent teeth, and that therefore any irregularities in the deciduous teeth should be corrected in order to prepare the way for the permanent teeth. If the deciduous teeth are brought to occupy a normal position, the crowns of the underlying permanent teeth will naturally take a correct position and their roots will be formed in harmony.—E. A. Bogue, New York, N. Y.

Seal all Dressings in Teeth With Cement.—Apply dressing in cavity, cover with ordinary card disc to prevent any pressure by cement coming in contact with dressing. Mix cement rather thin, so it will drop from spatula and adjust itself in cavity. This gives the patient an opportunity to use the tooth while under treatment, prevents their tasting medicine, and, lastly, you get the full benefit of the dressing without it becoming contaminated with the fluids of the mouth.—LeGrand M. Cox, St. Louis, Mo.

Edge Shape of Central Incisor.—After years of careful comparison I find that almost without any exception, the shape of the face turned upside down is the edge shape of the upper central incisor which belongs to that face. To state this more clearly, I will offer a few illustrations. We will imagine a line drawn across the forhead between the eyebrows, and hair line, and down each side of the face, cutting the crest of the check bone to the point of the chin. By inverting this we have the outline of an incisor tooth.—F. H. Berry, Dentist's Magazine.

Taking Impressions.—I have one little suggestion to offer in the taking of impressions—particularly in those cases that are extremely sensative and easily nauseated—that I have found useful and helpful, and that is to sponge the mouth with hydrogen dioxid. After thus having cleaned all the mucous surfaces apply a : 100 solution of eucain to the whole palate. That will enable one to take an impression in the most exaggerated cases of palatal sensitivity.—T. B. HARTZELL, Texas Dental Journal.

IMPRESSIONS.—That it is no longer necessary that women should lose a tooth for each child, as was the old belief That there is little ground for the belief that dental operations are dangerous to the pregnant woman. That dental care will improve the health of the mother. That the fetus is benefited by the dental care of the parent. That the child begins existence better equipped for the battle of life.—DR. M. E. JORDON, Era.

LOWER IMPRESSIONS.—The fitting of the tray for the lower impression is of more importance than that for an npper. The lower impression tray should be so bent that the tray shall be shallow at its posterior part on its buccal flanges, and deep enough on its lingual flanges to carry down between the tongue and the ridge. And it should be long enough to go back and cover the tuberosities. There should be about a quarter of an inch space between the lingual flanges of the tray and the lingual sides of the ridge, and should be so bent that the patient would be able, when the tray is in position, to thrust the tongue forward on top of the tray.—J. A. BULLARD, Review.

OBJECTION TO QUICK-SETTING AMALGAMS.—While the quick-setting alloy is an ideal material for saving teeth, yet on the other hand the quick-setting features are often the cause of amalgam failures. The operator endeavors to fill too many cavities with one mix, consequently the amalgam is used after partial setting has taken place. These fillings are lamentably weak in edge strength, and shrinkage ultimately prevails because the operator cannot work out the surplus mercury after it has chemically united with the metals of the alloy. At least two mixes should be made for large contour fillings in the event that quick-setting alloys are used.

Never Guarantee Work.—Never under any circumstances guarantee any operation. Give the public to understand that they are paying for professional services, not buying an article, and that you are giving them your best possible services. At the same time be courteous enough to rectify any mistakes or repair any work that might not have reached your ideal. The lawyer gets his fee whether he wins his case or not; likewise the physician whether his patient dies or recovers, both because they have given the best services to the case and did not guarantee anything. The public are too long used to the idea that they will pay for a piece of work when it fulfills their standard of a test.—Dr. S. D. Ruggles, Summary.

Dont's in Inlay Work.—Don't guess at the thickness of your matrix metal; use a micrometer.

Don't try to fill small cavities with gold inlays.

Don't use dirty borax.

Don't use anything for a flux except clean, freshly creamed borax.

Don't let the solder get through the matrix and on the back of the inlay.

Don't try to force your solder to flow with a small flame.

Don't smother your work with borax.

Don't use low grade solders.

Don't fill your inlays too full.

Don't expect too much of gold inlays and don't be discouraged by failures.—Dr. T. P. Hinman, Summary.

To Remove a Morbid Growth of Gum Tissue From a Cavity. —Frequently we have cases when a morbid growth of gum tissues fills, or partly fills, a cavity. Its removal is not only painful, but it is also a mean piece of work to cut it away on account of the excessive hemmorrhage. In the majority of such cases it can be removed neatly and with dispatch, painlessly and bloodlessly, by ligating with a piece of silk to either the tooth with the cavity or the adjoining one, which ever is the most convenient. This cuts off the blood supply and reduces to a minimum the pain when cutting away the growth with a side motion of a small flat burnisher or other suitable instrument. If a bad case, touch with trichloracetic acid. For those who have not tried this method of removing this troublesome growth the result will be surprising. Dental Office and Laboratory.

Glorious Gotham.—In New York. Every forty seconds an emigrant arrives. Every seven minutes there is a funeral. Every thirteen minutes a pair get married. Every forty-eight minutes a building catches fire. Every forty-eight minutes a ship leaves the harbor. Every fifty-one minutes a new building is erected. Every fifty-two seconds a passenger train arrives from some point outside the city limits. Every one and three-quarter hours some one is killed by accident. Every seven hours some one fails in business. Every eight hours an attempt to kill some one is made. Every eight and one-half hours some pair is divorced.—Ex.

No Ad., No Title. Physicians and dentists in the town of Lake Geneva Wis., have learned the lesson that even dignified and useful professions cannot flourish without some sort of advertising, says News paperdom. There is, as everybody knows, a hoary rule of "ethics" which forbids advertising by doctors, but in Lake Geneva it was for a long time honored in the breach. The physicians, and their brothers, the dentists, inserted cards in the local newspapers.

About two years ago an "ethical" revival took place in that burg. Smitten with a sense of grevious misconduct, the doctors stopped advertising. The local newspapers then quit calling them "Dr." on the perfectly sensible theory that the professional title is itself an advertisement. If all advertising is against the code, free ads, are as bad as paid ones. The newspapers, however, treated the doctors with fairness and courtesy. Beyond the suppression of the title there was no change in the papers' attitude toward them after they ceased to advertise. In time the townfolk dropped the "Dr." in their every-day talk and substituded "Mr." Recently the medical men have, like sensible men, seen the reason to change their views. They have discovered that a suitable card in the newspaper is no more undignified than an office sign. The cards are back in the papers and the doctors have ceased to be mere "misters."—Clinton Courant.

A METHOD FOR MAKING GOLD INLAYS WITHOUT A MATRIX.—After the decay has all been removed from the cavity, if extensive, fill with a quick-setting cement. Now prepare your cavity so that you will be absolutely sure that there are no under-cuts. Wrap a little cotton around a broach and moisten it with a slight lubricating oil. Protect the cavity from moisture with cotton, and wipe the cavity and portion of the tooth surrounding it with the broach moistened with the oil. Now pack your moss fiber gold into the cavity, being sure that all of the margins are covered perfectly. This can be done with hand pressure, and it is not desirable that there should be a high specific gravity. Burnish the gold perfectly over the margins, and insert a sharp instrument into the center of the filling and remove it. Cover the surface which would come into contact with the cavity with alcohol and rouge. Flow your solder to the desired contour and insert the filling and polish.

This will give you as perfect an adaptation as is possible to obtain with an inlay, also leaving a surface that the cement will adhere to much more perfectly than it would to the matrix, making an inlay with a better adaptation, better retentive qualities, and a saving of a great deal of time and inconvenience to the patient. This method I have followed for some time and find it entirely satisfactory.—ARTHUR E. PECK, M. D., D. D. S.

HOLLOW GOLD INLAY—.My method of procedure is as follows: I protect the adjoining tooth with Ivory's matrix band, when in close proximity to the adjoining tooth, then with chisels and with convex knife edged carborundum wheel slice off, and with burs form the cavity. This leaves a superficial cavity with margins beveled. The matrix may then be burnished into the cavity with very little trouble and as easily removed. I burnish directly into the tooth without regard to folds, annealing or tearing. It will be noticed that

I spend very little time with the matrix, which is never removed from the cavity until it is entirely finished. It is not necessary to imbed a platinum matrix for a gold inlay, but instead paint around the matrix with yellow ochre. This I find is stiffer and adheres better to the platinum than rouge. A small pellet of yellow ochre is placed in the center of the matrix over which the gold flows, giving the desired retaining form to the inlay. In this method I claim that the cavity is more easily and quickly prepared, and with less pain, the matrix is more easily formed and removed, the cavity is better protected and the inlay is so placed in relation to the tooth that the lines of force met with in mastication are directed centrally instead of extrinsically. In making an inlay I always use 22-24 carat gold—never a solder or flux.—E. L. Hutchinson, Honolulu, H. I.

